# Estimating High-Order Brain Functional Networks in Bayesian View for Autism Spectrum Disorder Identification

**DOI:** 10.3389/fnins.2022.872848

**Published:** 2022-04-27

**Authors:** Xiao Jiang, Yueying Zhou, Yining Zhang, Limei Zhang, Lishan Qiao, Renato De Leone

**Affiliations:** ^1^School of Mathematics Science, Liaocheng University, Liaocheng, China; ^2^School of Science and Technology, University of Camerino, Camerino, Italy; ^3^College of Computer Science and Technology, Nanjing University of Aeronautics, Nanjing, China; ^4^School of Computer Science and Technology, Shandong Jianzhu University, Jinan, China

**Keywords:** brain functional network, high-order network, Pearson’s correlation, Bayesian statistics, matrix-variate normal distribution, autism spectrum disorder

## Abstract

Brain functional network (BFN) has become an increasingly important tool to understand the inherent organization of the brain and explore informative biomarkers of neurological disorders. Pearson’s correlation (PC) is the most widely accepted method for constructing BFNs and provides a basis for designing new BFN estimation schemes. Particularly, a recent study proposes to use two sequential PC operations, namely, correlation’s correlation (CC), for constructing the high-order BFN. Despite its empirical effectiveness in identifying neurological disorders and detecting subtle changes of connections in different subject groups, CC is defined intuitively without a solid and sustainable theoretical foundation. For understanding CC more rigorously and providing a systematic BFN learning framework, in this paper, we reformulate it in the Bayesian view with a prior of matrix-variate normal distribution. As a result, we obtain a probabilistic explanation of CC. In addition, we develop a Bayesian high-order method (BHM) to automatically and simultaneously estimate the high- and low-order BFN based on the probabilistic framework. An efficient optimization algorithm is also proposed. Finally, we evaluate BHM in identifying subjects with autism spectrum disorder (ASD) from typical controls based on the estimated BFNs. Experimental results suggest that the automatically learned high- and low-order BFNs yield a superior performance over the artificially defined BFNs *via* conventional CC and PC.

## Introduction

Resting-state functional magnetic resonance imaging (rs-fMRI)-based brain functional network (BFN) analysis without a specific task, has shown a great potential to discover biomarkers for identifying neurological/mental disorders, such as autism spectrum disorder (ASD) (Wang et al., 2019), major depressive disorder (MDD) (Long et al., 2020), schizophrenia (Ariana and Cohen, 2013), Parkinson’s disease (PD) (Baggio et al., 2014), Alzheimer’s disease (AD) (Hahn et al., 2013), and its early stage, namely, mild cognitive impairment (MCI) (Jiang et al., 2019). However, the identification of brain disorders based on the BFN remains a critical challenge, since its great performance depends on multiple interactive factors including reasonable brain parcellation, well-parametrized network estimation, discriminative feature selection/extraction, and powerful classifier design (Dadi et al., 2019; Pervaiz et al., 2020). Instead of considering all these aspects that have been empirically evaluated in recent studies (Dadi et al., 2019; Pervaiz et al., 2020), in this paper, we mainly focus on the BFN estimation issue. Recently, more advanced studies (Yu et al., 2017; Zhang et al., 2017; Mahjoub et al., 2018) have proposed the brain functional connectivity representations for estimating BFN at different connectivity levels, including low-order, high-order, etc. Low-order methods are designed to characterize the synchronization of blood oxygen level dependent (BOLD) signals and are insufficient to characterize a high level of interaction. Recent literature (Chen et al., 2016; Zhang et al., 2016) presented high-order methods to measure the relationship between the BFN connectivity. This paper specifically aims to capture the brain connectivity that is supposed to exist in a higher-order form.

Owing to its non-invasiveness and easy reproducibility, rs-fMRI (Wang et al., 2020) has become a widely used technique to estimate BFN whose nodes correspond to spatial regions of interest (ROIs) and edges describe the relationship (e.g., similarity, correlation, synchronism, etc.) between the rs-fMRI signals associated with these ROIs. In the past decades, researchers have developed many BFN estimation methods, including Pearson’s correlation (PC) (Biswal et al., 1995; Eguiluz et al., 2005), partial correlation (Marrelec et al., 2006), regularized full/partial correlation (Friedman et al., 2008; Jie et al., 2009; Li et al., 2017), structural equation modeling (Mclntosh and Gonzalez-Lima, 1994), and dynamic causal modeling (Friston et al., 2003), etc. According to a recent comparative study (Smith et al., 2013), the correlation-based approaches are “quite successful” for estimating informative BFNs. Particularly, PC is the fundamental and most widely used correlation-based method for BFN estimation. Despite its empirical effectiveness, PC only considers a pair of ROIs at a time, and thus suffers the confounding effect from other ROIs. Partial correlation can tackle this problem by regressing out the confounding variables. However, that may lead to an ill-posed estimation since the partial correlation is usually calculated by inverting a covariance matrix that may be singular. In practice, a regularizer is generally introduced into the partial correlation model, which not only deals with the ill-posed problem but also provides a natural way to introduce topological priors of the brain network into the estimation models. Specifically, L_1_-norm is commonly used to encode the sparsity prior of the BFN (Lee et al., 2011), a weighted version of the L_1_-norm to capture the hub structure (prior) (Li et al., 2017), the L_2,1_-norm to model group sparsity (or population prior) that imposes all the subjects share the same BFN topology (Zhang, 2010), and a combination of L_1_-norm with trace-norm to encode the modularity (prior) of the BFN (Qiao et al., 2016), to just name a few.

No matter which prior or regularizer is introduced, most of the correlation-based methods only estimate low-order BFNs whose edges are the full or partial correlation of the rs-fMRI time series. Beyond these traditional low-order correlations, researchers found that some forms of high-order correlations may contain useful feature information for BFN analysis and classification (Chen et al., 2016; Zhang et al., 2016; Zhou et al., 2018a,b). For example, Chen et al. (2016) defined the high-order correlation as the dependency between functional connectivity fluctuation, with clustered mean correlation time series as input. Different from characterizing a temporal correlation, Zhang et al. (2016) proposed to construct the high-order BFN to examine spatial properties of the functional connectivity network. Specifically, such a scheme is achieved by two sequential PC operations, where the first PC operation is used to construct a traditional low-order BFN, and the ensuing PC operation is conducted on the edge weights of the estimated BFN to generate the high-order BFN. Despite encoding the network information from different dimensions, the above methods are uniformly called correlation’s correlation (CC) (Zhang et al., 2017) since they both involve two PC operations in the high-order BFN construction. However, the CC-based high-order BFNs are estimated intuitively and heuristically without the support of any strong theoretical basis.

Toward a better understanding of CC, in this paper, we reformulate it in the Bayesian framework with a prior that the low-order BFN follows the matrix-variate normal (MVN) distribution. As a result, we obtain a probabilistic explanation for CC and develop a new method that both learns low- and high-order BFNs from data based on the rigorous theoretical framework. In brief, we summarize the main contributions of this paper as follows.

1. We reformulate PC from a statistical point of view. Based on this, a regularized statistical framework is derived by introducing Gaussian distribution to the error term, which provides a more flexible modeling idea.
2. A mathematical model for a high-order learning method based on CC is developed by assuming the adjacency matrix of low-order brain networks follows an aprior normal distribution.
3. Based on the probabilistic framework derived above, an automatic learning model, namely, BHM, is proposed. Compared with the traditional high-order network learning method (i.e., CC), the model simultaneously learns low-order and high-order brain networks. In the learning process, the direct information of the low-order network and the indirect information of the high-order network complement each other toward more reliable/discriminative brain networks.
4. Finally, we empirically verify that the automatically learned BFNs outperform the artificially defined ones *via* CC and other baselines in the identification of ASD, even with a simple feature selection method and classifier.

For a consistent expression throughout the paper, we first describe the basic notations as follows. Scalars involving the variables, parameters, and constants are denoted by *italic* lowercase letters, e.g., *x*. Vectors are denoted by bold lowercase letters and the elements inside are stored in a column, e.g., **x** = (*x*_1_, *x*_2_, ⋯, *x*_*n*_)^*T*^. Matrices are denoted by bold uppercase letters such as **X**.

The rest of the paper is organized as follows. In Section “Related Works,” we review the related works including PC, sparse representation (SR), and CC. In Section “High-Order Correlation Learning,” we first introduce a theoretical framework for explaining CC and then develop a new framework for learning high-order BFN by reformatting CC in a view of the maximal posterior probability. In Section “Experiments and Results,” we conduct experiments to evaluate the discrimination of the automatically learned high-order BFNs. In Section “Discussions,” we discuss the main findings. Finally, the conclusion are reported in Section “Conclusion.”

## Related Works

In this section, we review three related works: PC, SR, and CC. As discussed previously, PC and SR are used to construct the traditional **low-order** BFNs, while CC as a two-step sequential PC method is used to estimate the **high-order** BFNs.

### Pearson’s Correlation

Suppose **x**_*i*_ is the multivariate random variable (random vector) associated with the *i^th^* ROI. Then, the observed rs-fMRI signals^1^ **x**_*i*_ = (*x*_1*i*_, *x*_2*i*_, ⋯, *x*_*ni*_)^*T*^, *i* = 1, 2, ⋯, *p* can be considered as a sampling of the multivariate random variable (or population) **x**_*i*_, where *p* is the number of ROIs and *n* is the number of time points. Since our goal is to estimate the edge weights of the BFN, the simplest and empirically effective way is to calculate the sample PC coefficient ${\hat{w}}_{ij}\left(i,j=1,2,\cdots ,p\right)$ of pair-wise ROIs, as follows.


(1) $${\hat{w}}_{ij}=\frac{{\left(x_{i}-{\bar{x}}_{i}\right)}^{T}\left(x_{j}-{\bar{x}}_{j}\right)}{\sqrt{{\left(x_{i}-{\bar{x}}_{i}\right)}^{T}\left(x_{i}-{\bar{x}}_{i}\right)}\sqrt{{\left(x_{j}-{\bar{x}}_{j}\right)}^{T}\left(x_{j}-{\bar{x}}_{j}\right)}}$$

where ${\bar{\text{x}}}_{i}$ is the mean vector corresponding to **x**_*i*_. Under Gaussian assumption, Eq. 1 gives an asymptotically unbiased estimation for the population PC coefficient. Without loss of generality, we redefine ${\text{x}}_{i}≜\left({\text{x}}_{i}-{\bar{\text{x}}}_{i}\right)/\sqrt{{\left({\text{x}}_{i}-{\bar{\text{x}}}_{i}\right)}^{T}\left({\text{x}}_{i}-{\bar{\text{x}}}_{i}\right)}$. Then, the estimator of population PC coefficient can be simplified as follows:


(2) $${\hat{w}}_{ij}={\text{x}}_{i}^{T}{\text{x}}_{j}or\hat{\text{W}}={\text{X}}^{\text{T}}\text{X}$$

where **X** = [**x**_1_, **x**_2_, ⋯, **x**_*p*_] is the rs-fMRI data matrix whose columns are the rs-fMRI time series associated with different ROIs. Therein, $\hat{\text{W}}$ is the generalized estimator in matrix form.

### Sparse Representation

Sparse representation is one of the commonly used methods for calculating partial correlation among ROIs. A regularization term encoding sparsity prior is introduced into the BFN

estimation model. Specifically, the mathematical model of SR is given by:


(3) $$\min_{W}\sum_{i=1}^{p}{||{\text{x}}_{i}-\sum_{j\neq i}w_{ij}{\text{x}}_{j}||}^{2}+\lambda \sum_{j\neq i}\left|w_{ij}\right|$$

where **W** is the edge weight matrix of BFN.

Similar to PC, we can rewrite SR in matrix form:


$$\min_{W}{||\text{X}-\text{XW}||}_{F}^{2}=\lambda {||W||}_{1}$$

(4) $$s.t.w_{ii}=0,\forall i=1,2,\cdots ,p$$

where the constraint *w*_*ii*_ = 0 plays a role in removing **x**_*i*_ from **X** to avoid the trivial solution.

### Correlation’s Correlation

Despite its popularity and effectiveness, the traditional PC can only construct the low-order BFN. That is, the connection between two ROIs is determined by the correlation of the corresponding rs-fMRI time series. However, in practice, a connection can be described in *both* low-order *and* high-order views. For example, we can directly define a connection between ROI *i* and *j* if there is a relationship. Besides, if ROIs *i* and *j* are connected to the same brain region, we can infer with a great possibility that there is a connection between ROI *i* and *j*. The former corresponds to the correlation in the traditional low-order view, while the latter can be considered as CC in a high-order perspective. This results can be achieved by a two-step procedure. First, the low-order BFN is estimated *via* PC. According to the formula in Eqs 1 or 2, the adjacency matrix $\hat{\text{W}}={\left({\hat{w}}_{ij}\right)}_{p=p}$ of the PC-based BFN can be calculated as follows.


(5) $${\hat{h}}_{ij}={\hat{\text{w}}}_{i}^{T}{\hat{\text{w}}}_{j}$$

where ${\hat{\text{w}}}_{i}$ and ${\hat{\text{w}}}_{j}$ are the *i^th^* and *j^th^* columns of $\hat{\text{W}}$, respectively. For simplicity, in Eq. 5, ${\hat{\text{w}}}_{i}$ and ${\hat{\text{w}}}_{j}$ has been centralized and normalized as the case in Eq. 2. As a result, the CC-based high-order BFN is defined as follows,


(6) $$\hat{\text{H}}={\left({\hat{h}}_{ij}\right)}_{p\times p}={\hat{\text{W}}}^{T}\hat{\text{W}}$$

## High-Order Correlation Learning

As described previously, CC constructs the high-order BFN based on two sequential correlation operations. Despite its empirical effectiveness in identifying neuro-disorders (Chen et al., 2016; Zhang et al., 2016), CC is a measure defined intuitively without a clear mathematical/probabilistic explanation. Therefore, in this section, we will construct a more rigorous mathematical model for CC, in order to provide a better understanding of the CC-based high-order BFN.

Since CC is based on the PC variant, in the following Section “Pearson’s Correlation-Based Brain Functional Network Learning Framework in Bayesian View,” we first reformulate PC into a more flexible BFN estimation framework. Then, based on the framework, we establish a theoretical model for CC in Section “Learning High-Order Brain Functional Network With a Matrix-Normal Penalty.” Finally, we design an algorithm for learning the high-order BFN based on the theoretical model of CC in Section “Algorithm”.

### Pearson’s Correlation-Based Brain Functional Network Learning Framework in Bayesian View

As we know, PC is a measure of the linear correlation between pair-wise rs-fMRI time series associated with the ROIs. In other words, a time series **x**_*i*_ = (*x*_1*i*_, *x*_2*i*_, ⋯, *x*_*ni*_)^*T*^ can be linearly represented by other time series **x**_*j*_ = (*x*_1*j*_, *x*_2*j*_, ⋯, *x*_*nj*_)^*T*^ as


(7) $$x_{i}=a_{ij}x_{j}+\varepsilon_{i}$$

where *a*_*ij*_ is the representative coefficient, and *ε*_*i*_ = (ε_1*i*_, ε_2*i*_, ⋯, ε_*ni*_)^*T*^ is the random error vector. That is, for each variable, we have:


(8) $$x_{ki}=a_{ij}x_{kj}=\varepsilon_{ki}\left(k=1,2,\cdots ,n;;i,j=1,\cdots ,p\right)$$

Generally, we assume that the random variable ε_*ki*_ follows a normal distribution with mathematical expectation 0, i.e., ε_*ki*_∼𝒩(0, σ^2^). Therefore, given *x*_*kj*_ and *a*_*ij*_ are constants, *x*_*ki*_ follows the normal distribution *x*_*ki*_∼𝒩(*a*_*ij*_*x*_*kj*_, σ^2^). Then, The following formula can be obtained by the maximum likelihood estimation of *x*_*ki*_ (see Appendix A for details).


(9) $$\max_{a_{ij}}-n\log\left(\sqrt{2\pi \sigma }\right)-\frac{{||{\text{x}}_{i}-a_{ij}{\text{x}}_{j}||}^{2}}{2\sigma^{2}}$$

Note that Eq. 9 can be equivalently written as the following least-squares problem:


(10) $$\underset{a_{ij}}{\text{min}}{||{\text{x}}_{i}-a_{ij}{\text{x}}_{j}||}^{2}$$

The optimal solution to Problem (10) is given by ${\hat{a}}_{ij}={\left({\text{x}}_{j}^{T}{\text{x}}_{j}\right)}^{-1}{\text{x}}_{j}^{T}{\text{x}}_{i}={\text{x}}_{i}^{T}{\text{x}}_{j}={\hat{w}}_{ij}$, considering that all of time series **x**_*i*_, *i* = 1, 2, ⋯, *p* have been normalized by $\frac{x_{i}}{||x_{i}||}$. This means that the solution of Eqs 9, 10 is the same as PC shown in Eq. 2. Therefore, in what follows, we only use *w*_*ij*_ instead of *a*_*ij*_ for the consistency of mathematical notations.

To provide a more flexible framework for BFN estimation, we further generalize PC in Bayesian view by introducing a prior distribution on *w*_*ij*_. Although various distributions can be used as the prior, here we first consider the standard normal distribution, i.e., *w*_*ij*_∼𝒩(0,1), since it provides a basis for understanding more complex cases. However, in practice, the entries *w*_*ij*_ in **W** may not be apriori independent of each other, but exist a relationship. Due to *w*_*ij*_∼𝒩(0,1), the edge weight *w*_*ij*_ of BFN has the following prior probabilistic density:


(11) P(wij)=12πe-wij22

Next, a maximal posterior estimation of *w*_*ij*_ (see Appendix B for details) can be obtained as follows,


(12) maxwij-(n+1)log⁡(2π)=(-n)log-||xi-wijxj||2+σ2wij22σ2

The above problem is equivalent to the regularized least-squares problem:


(13) $$\min_{w_{ij}}{||{\text{x}}_{i}-w_{ij}{\text{x}}_{j}||}^{2}+\lambda w_{ij}^{2}$$

where λ = σ^2^ in the case of standard normal distribution. In practice, λ≜σ2/σ02 is a hyper-parameter that controls the balance between the two terms in Eq. 13, where σ02 corresponds to the variance of normal distribution of the edge weight *w*_*ij*_. Setting the gradient of the objective function to zero, we obtain the optimal solution of Eq. 13 as follows:


(14) $${\hat{w}}_{ij}={\left({\text{x}}_{j}^{T}{\text{x}}_{j}+\lambda \right)}^{-1}{\text{x}}_{j}^{T}{\text{x}}_{i}={\left(1+\lambda \right)}^{-1}{\text{x}}_{i}^{T}{\text{x}}_{j}$$

We find that it is a shrinkage of the original estimation of PC which helps remove the weak connections in BFN.

Note that Eq. 13 only considers finding one edge weight at a time. Without loss of generality, with the assumption that the variables *w*_*ij*_ in **W** are independent, we can rewrite Eq. 13 in the following matrix form:


(15) $$\min_{W}{||\text{W}-{\text{X}}^{T}\text{X}||}_{F}^{2}+\lambda tr\left({\text{WW}}^{T}\right)$$

where $tr\left({\text{WW}}^{T}\right)=\sum_{i,j}w_{ij}^{2}$ is the trace operator of **WW**^*T*^. As a result, we achieve the estimation of BFN in a batching way as follows,


(16) $$\hat{\text{W}}={\left(1+\lambda \right)}^{-1}{\text{X}}^{T}\text{X}$$

This formula is essentially a generalization of Eq. 2 with a shrinkage factor (1+λ)^−1^. When λ+0, Eq. 16 reduces to the traditional PC.

### Learning High-Order Brain Functional Network With a Matrix-Normal Penalty

**FIGURE 1:**
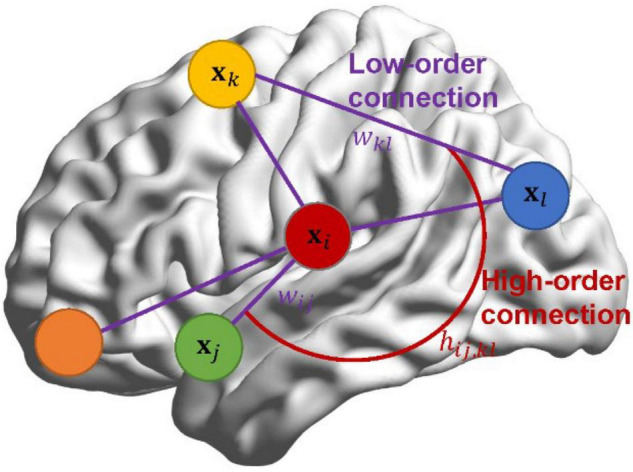
The diagram of low- and high-order connections.

In Section “Pearson’s Correlation-Based Brain Functional Network Learning Framework in Bayesian View,” we reformulate PC and then generalize it in Bayesian view by introducing a standard normal prior *w*_*ij*_∼𝒩(0,1) for each pair of ROIs (*i*, *j*), *i*, *j* = 1, 2, ⋯, *p*. However, in practice, the entries *w*_*ij*_ in **W** may not be apriori independent of each other, but exist a relationship. Even so, Section “Pearson’s Correlation-Based Brain Functional Network Learning Framework in Bayesian View” provides a flexible probabilistic framework to develop new brain network estimation methods. Inspired by this point, we lay down theoretical support for CC from the Bayesian perspective. More importantly, instead of assuming that *w*_*ij*_ in **W** are independent, we propose a Bayesian high-order model (BHM) for BFN estimation by introducing the prior of matrix-variate normal distribution to the low-order BFN **W**. BHM learns the high-order relationship from the data automatically, rather than manually define as the case in CC. Interestingly, such a scheme can simultaneously learn low-order and high-order BFNs by considering the spatial structure of network connections. To distinguish the low-order and high-order correlations, an illustration is shown in Figure 1. The connections among *w*_*ij*_ can be considered as a high-order correlation *h*_*ij,kl*_, while *w*_*ij*_ denotes the traditional low-order linear correlation between ROIs.

#### Model

Since we can vectorize the low-order edge weight matrix **W** into a *p*^2^ = 1 vector, the low- and high- order correlation can be modeled by a multivariate normal distribution, *vec*(**W**∼)𝒩(**O**, **Ω**), where **W** encodes the low-order relationship and **Ω** ∈ *R*^*p*^2^ = *p*^2^^ is the covariance matrix for modeling the relationship between the entries in **W**. Despite the theoretical feasibility for encoding the high-order relationship, **W**-vectorization ignores its spatial structure as a matrix. Even worse, the estimation of **Ω** is extremely challenging due to its high dimension. Such a scale *not only* goes beyond the storage ability of the general memory (Specifically, in our experiment, *p* is 160, which takes up about 4.9 GB storage), *but also* may lead to the overfitting problem. Therefore, we further assume that the covariance matrix **Ω** has the Kronecker product decomposition (Gupta and Nagar, 2000), i.e., **Ω** = **Ω**_1_⊗**Ω**_2_, where **Ω**_1_ and **Ω**_2_ denote the **row** and **column** covariance matrices, respectively. That is, **W** follows the distribution **W**∼ℳ𝒩(**O**, **Ω**_1_⊗**Ω**_2_). As described earlier, in this paper, we mainly focus on correlation-based methods that generally result in the symmetric BFN. Therefore, the row and column covariance matrices of **W** are the same, i.e., **Ω**_1_ = **Ω**_2_, and without loss of generality, we define **Ω**≜**Ω**_1_ = **Ω**_2_. As a result, the matrix-variate normal distribution (Gupta and Nagar, 2000) of the low-order BFN **W** has a probability density:


(17) $$P\left(W\right)={\left(2\pi \right)}^{-\frac{1}{2}p^{2}}{\left|\Omega \right|}^{-p}e^{tr\left\{-\frac{1}{2}\Omega^{-1}W\Omega^{-1}W^{T}\right\}}$$

Similar to the formulation in Eqs 11–16, we take Eq. 17 as a prior distribution of low-order network **W**. Then, we can formulate the posterior probability of **W** based on the Bayesian rule (see Appendix C for details). By maximizing the posterior probability, the low- and high-order BFN mutual learning model can be obtained as follows:


(18) $$\begin{array}{c} J\left(W,\Omega \right)=\min_{W,\Omega }||W-{\text{X}}^{T}\text{X}|{|}_{F}^{2}+\lambda [\frac{1}{2}tr\left(\Omega^{-1}W\Omega^{-1}W^{T}\right)\\ \;+p\log\left(|\Omega |\right)] \end{array}$$

where **W** is the Bayesian low-order BFN, **Ω** corresponds to the Bayesian high-order BFN, *p* is the number of ROIs, and λ is a hyper-parameter that controls the balance between the two terms in the objective function.

#### Algorithm

The alternating optimization (AO) scheme (Bezdek and Hathaway, 2002) is employed to solve Problem (18). More specifically, we first initialize the low-order BFN using the PC estimator, i.e., $\text{W}=\hat{\text{W}}={\text{X}}^{T}\text{X}$, and then alternatively optimize **W** and Ω.

**Step 1** Fix **W** and solve **Ω**. The optimization problem is


(19) $$\min_{\Omega }tr\left(\Omega^{-1}\text{W}\Omega^{-1}{\text{W}}^{T}\right)+p\text{log}\left(\left|\Omega \right|\right)$$

which can be solved by the following iterative formula (Dutilleul, 1999; Zhang and Schneider, 2010)


(20) $$\Omega ={\text{W}}^{T}\Omega^{-1}\text{W}$$

Note that, by initializing **Ω** = **I** in Eq. 20, at the first iteration, we obtain **Ω** = **W**^T^**W**, which reduces to the traditional CC, as $\hat{\text{H}}$ defined in Eq. 6. In other words, the traditional CC is only a rough estimation of the theoretical value at the first iteration. We can continue the iteration toward a more accurate estimation of **Ω**. In fact, with the estimated **Ω**, we can further update **W** according to the AO scheme. In practice, we generally add a small quantity δ**I** to Eq. 20 for a more stable numerical solution where δ is a small positive constant.

**Step 2** Fix **Ω** and solve **W**. The optimization problem is


(21) $$\min_{W}{||\text{W}-{\text{X}}^{T}\text{X}||}_{\text{F}}^{2}+\frac{\lambda }{2}tr\left(\Omega^{-1}\text{W}\Omega^{-1}{\text{W}}^{T}\right)$$

With the fixed **Ω**, the gradient of Eq. 21 with respect to **W** is


(22) $$2\text{W}-2{\text{X}}^{T}\text{X}+2\lambda \Omega^{-1}\text{W}\Omega^{-1}$$

Setting the gradient equal to zero, we obtain:


(23) $$\text{W}={\text{X}}^{T}\text{X}-\Omega^{-1}\text{W}\Omega^{-1}$$

We summarize the algorithm for solving Problem (18) in Algorithm 1.

**Algorithm 1: Estimating BFN with BHM model.**

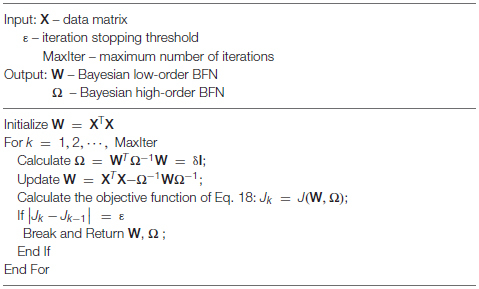

## Experiments and Results

### Data Acquisitions and Processing

To evaluate the effectiveness of the proposed BHM, we conduct experiments on Autism Brain Imaging Data Exchange (ABIDE) database. The objective is to identify subjects with ASD from typical controls (TCs). Considering the heterogeneity of multi-site data, we only use data from the *NYU* site in our study. The dataset includes 184 subjects (79 ASD patients and 105 TCs). The detailed scan procedures and protocols are described on the ABIDE website.^2^ The demographic information of all participants is summarized and displayed in Table 1.

**TABLE 1:** Demographic information of the used dataset.

	ASD (*n* = 79)	TC (*n* = 105)
Gender (M/F)	68/11	79/26
Age (year SD)	14.51 ± 6.23	15.80 ± 3.23
FIQ (mean SD)	107.91 ± 16.62	113.15 ± 13.12
ADOS (mean SD)	11.3 ± 4.08	–

*ASD, autism spectrum disorders; TC, typical control; FIQ, full intelligence quotient; ADOS, autism diagnostic observation schedule.*

All rs-fMRI images were acquired using a standard echo-planar imaging sequence on a clinical routine 3T Siemens Allegra scanner. During the 6-min rs-fMRI scanning procedure, most subjects were required to relax with their eyes focusing on a white fixation cross in the middle of the black background screen projected on a screen. A few participants close their eyes. The functional scan parameters include the flip angle = 90°, 33 slices, TR/TE = 2000/15 ms with 180 volumes, FOV = 240 mm and voxel size = 3 ×3 ×4 mm^3^. The rs-fMRI data were preprocessed by DPARSF^3^ software. Specifically, to avoid the interference of early signal instability, the first 5 rs-fMRI volumes of each subject were discarded. The remaining volumes were calibrated as follows: (1) Slice timing correction and head motion correction; (2) Regression of nuisance signals (ventricle, white matter) and head-motion with Friston 24-parameter model (Friston et al., 1996); (3) Normalization and register to MNI space with resolution of 3 3 3 mm^3^; (4) Segmentation using DATTEL; (5) Spatial smoothing by a kernel of 6 mm. After that, since our focus is functional connectivity, the rs-fMRI time series signals were partitioned into 160 ROIs, according to the functional atlas Dosenbach 160 (Dosenbach et al., 2010). Finally, the mean time series of the ROI were put into a data matrix **X** ∈ *R*^175=160^, which will be used for the subsequent BFN estimation.

### Brain Functional Network Construction, Feature Selection, and Classification

With the preprocessed rs-fMRI data, we estimate the low- and high-order BFNs using the proposed method, i.e., Bayesian low-order Network **W** and Bayesian high-order Network **Ω**, respectively. For comparison, we also choose PC, SR, and traditional CC as baseline methods to construct BFNs.

Once the BFNs are constructed, the next step is feature selection and classification. In our study, we directly use edge weights of the estimated BFN as features for ASD identification. Despite its simplicity (without complex feature design), such a scheme easily causes the curse of dimensionality due to limited sample size. As described previously, the number of ROIs is 160 and thus the estimated feature edges are 160=(160−1)/2=12720, which is far greater than the sample size (i.e., the number of subjects 184). To alleviate the problem of small sample size, we adopt a two-sample *t*-test with an empirically fixed *p* values to select features before ASD classification. In our experiments, we evaluate five candidate parametric values of *p*, that is [0.001,0.005,0.01,0.05,0.1]. The specific parameter analysis results are given in Section “Sensitivity to Network Modeling Parameters.”

To perform the following classification task, we use a linear support vector machine (SVM) (Chang and Lin, 2011) with default *C* = 1 as the classifier. To evaluate the model, we adopt leave-one-out cross-validation (LOOCV) in our experiments due to the limited data samples. Specifically, a LOOCV works in each run and only one sample is used to test while the rest are used to train a classifier. The final performance is obtained by the averaged results of all the runs. Note that the model parameters are involved in certain methods, including SR and BHM. Therefore, we additionally adopt an inner LOOCV procedure on the training data to obtain the optimal parametric value. Specifically, for SR, the regularization parameter λ is set to [2^−2^, 2^−1^, 2^0^, 2^1^, 2^2^]. For the proposed BHM, the regularization parameter λ is set to [0.0001, 0.001, 0.01, 0.1, 1]. To be consistent with the number of parameters in other methods, the coefficient δ of the perturbation involved in Eq. 20 is set to 0.1 empirically.

### Classification Results

To evaluate the classification results of different methods, we use accuracy (ACC), sensitivity (SEN), specificity (SPE) as performance metrics. The definition of these quantities are reported in Table 2. Note that, in this work, we treat ASD patients as the positive class while the NCs as the negative class.

**TABLE 2:** Different performance metrics.

Performance metrics	Abbreviations	Definitions
Accuracy	ACC	$\frac{\text{TP}+\text{TN}}{\text{TP}+\text{FP}+\text{TN}+\text{FN}}$
Sensitivity	SEN	$\frac{\text{TP}}{\text{TP}+\text{FN}}$
Specificity	SPE	$\frac{\text{TN}}{\text{TN}+\text{FP}}$

*TP, TN, FP, and FN indicate true positive, true negative, false positive, and false negative, respectively.*

In Table 3, we report the ASD classification results of five methods. It can be observed that the Bayesian low-order network (BHM-**W**) and Bayesian high-order network (BHM-**Ω**) constructed by the proposed BHM perform better than the BFNs constructed by the traditional PC and CC, respectively. Moreover, BHM-**Ω** achieves the best performance. Besides, the high-order BFNs (traditional CC and BHM-**Ω**) are associated with better recognition performance when they are compared with the baseline methods PC, SR, and BHM-**W**. This means that the high-order network structure can provide more helpful information for BFN analysis to some extent. Furthermore, for two corresponding low-order methods, the performance of the traditional PC and BHM-**W** are approximately similar whereas BHM-**W** has slightly better accuracy than the traditional PC. This may benefit from the guidance information provided by the Bayesian high-order network **Ω** in the optimization process.

**TABLE 3:** The classification results based on five different methods for ASD identification.

Methods	ACC	SEN	SPE
PC	0.6359	0.6222	0.6383
SR	0.6033	0.2658	0.8571
CC	0.6630	0.5570	0.7429
BHM-**W**	**0.6576**	**0.5316**	**0.7143**
BHM-**Ω**	**0.7283**	**0.6329**	**0.8000**

## Discussion

### Brain Functional Network Visualization

**FIGURE 2:**
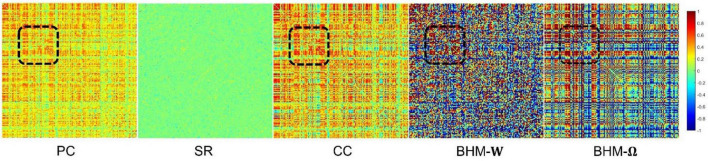
The BFN adjacency matrices of different methods. The patches marked by the black box are the consistent part of the network constructed by different methods.

**FIGURE 3:**
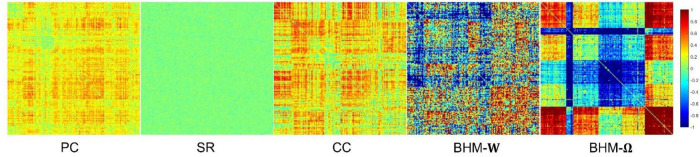
Five clustered edge weight matrices of the same subject estimated by different methods.

To evaluate the BFNs estimated by different methods, we randomly select a subject and visualize the BFNs constructed by PC, SR, CC, BHM, as shown in Figure 2. Specifically, the different colors of Figure 2 indicate different weights of the edge weights matrix (i.e., the BFN), ranging from −1 to 1. It is observed that: (1) Compared with SR, the BFNs estimated by the correlation-based methods (i.e., PC, CC, BHM) are denser since the sparsity prior is introduced into SR. (2) There are fewer areas of cold colors in the PC network heatmap, implying that the edges with the negative weights are less. (3) Compared with PC, CC’s network heatmap shows a sharper distinction between the areas of warm and cold colors, indicating that the positive edge weights of CC’s BFN are larger and the negative edge weights are smaller. (4) The BHM-**W** estimated from the Bayesian perspective has a greater distinction between positive and negative edge weights than PC. (5) The BHM-**Ω** as a Bayesian version of the traditional CC tends to produce a greater distinction between positive and negative edge weights than CC. Combined with the fact that the high classification accuracy of the BHM method, we can infer that the negative edge weights of BFN also have important information for classification. (6) The BFNs based on the correlation methods show a degree of consistency., as shown in the black box in Figure 2. Similar structures appear in the four BFNs estimated by PC, CC, BHM, which can provide certain support for the reliability of the three correlation methods. In addition, we cluster the brain regions using spectral clustering (Ng et al., 2001) and visualize the clustered adjacency matrices of these 5 methods in Figure 3. It is observed that the BFN estimated by BHM-**Ω** shows a more significant modular structure.

### Sensitivity to Network Modeling Parameters

**FIGURE 4:**
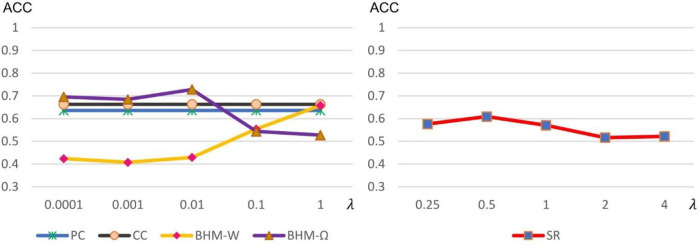
Classification accuracy of ASD identification based on 5 BFNs estimated by PC, CC, SR, and BHM with 5 different parametric values. Although PC and CC have no optional parameters, in order to facilitate comparison, we visualize the accuracy of PC and CC in the left chart.

As stated earlier, some methods including SR and BHM involve optional model parameters. Different parameter values may have a significant impact on the results. Therefore, we calculate the accuracy of different methods under different parameter values, as shown in Figure 4. It is worth noting that, traditional PC and CC models do not involve optional parameters. However, for comparison, we fix their values with the final classification accuracy in Figure 4 for visualization. We can observe that BHM-**Ω** and BHM-**W** are quite sensitive to the parameters. When λ in BHM is set to a large real number, the accuracy of BHM-**Ω** decreases, which may be because the large value of λ, the algorithm has difficulties to converge. Besides, the accuracy of BHM-**W** increases as λ increases. For this, we empirically tested a larger lambda range [2^−5^, 2^−4^, ⋯, 2^0^, ⋯, 2^4^, 2^5^] and find that as λ continues to increase, the accuracy decreases, which is consistent with the performance of BHM-**Ω**. Moreover, SR is not sensitive to different parameter values, but its accuracy performs average in general.

**FIGURE 5:**
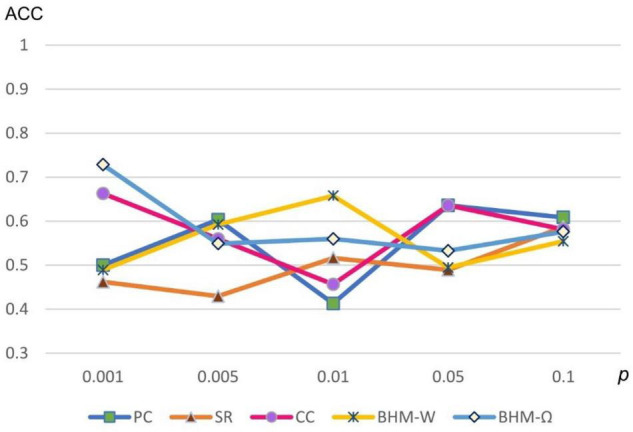
Classification accuracy of ASD identification based on 5 BFNs estimated by PC, CC, SR, and BHM for 5 different *p*-values.

Considering that different *p*-values significantly influence the results, we show the classification accuracies of 5 methods under different *p*-values in Figure 5. Note that all 5 methods are sensitive to different *p*-values. We selected the optimal parameter value for feature selection, so that different methods can get the best classification performance.

### Top Discriminative Features

**FIGURE 6:**
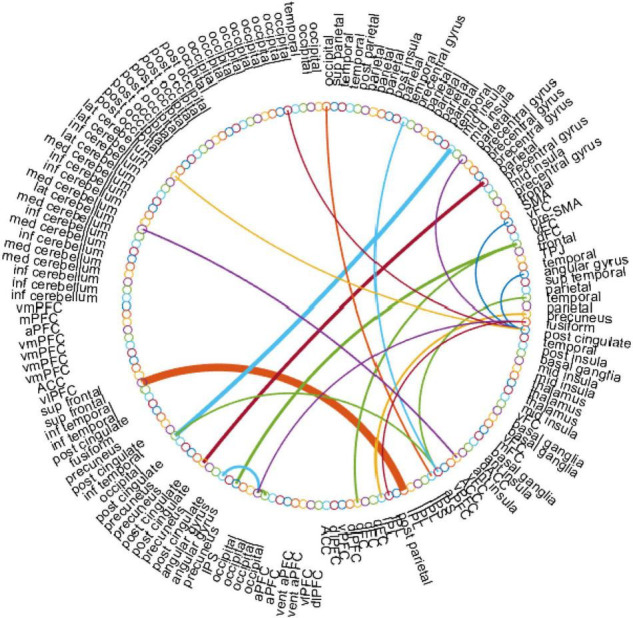
The most discriminative edge features of the BHM-**Ω** involved in the ASD classification task by using a *t*-test (*p* < 0.001). This figure is created by the circularGraph tool, which is designed by Paul Kassebaum and can be downloaded from http://www.mathworks.com/matlabcentral/fileexchange/48576-circulargraph.

In this work, for the ASD classification task, we use the edge weights of the estimated BFN as features. With the empirically optimal parameter, we construct the BFNs using the proposed BHM, then apply a two-sample *t*-test to rearrange the features according to the *p*-values. Particularly, we choose the BHM-**Ω** since it outperforms the BFNs estimated by the other methods. As a result, we obtain the discriminative edge connections with a threshold value *p* < 0.001 as shown in Figure 6. Here, the thickness of each arc represents the discriminative power that is inversely proportional to the corresponding *p*-value. The colors of each arc are assigned randomly for better visualization.

**FIGURE 7:**
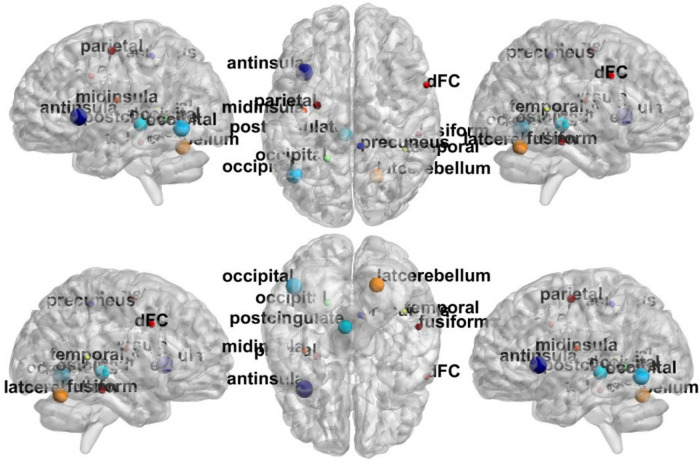
The full view of most relevant ROI associated with the ASD classification task based on BHM-Ω. This visualization is created using the BrainNet Viewer (https://www.nitrc.org/projects/bnv/).

In Figure 6, the top discriminative features and the corresponding brain regions, that may contribute to ASD identification include occipital lobe, post-cingulate, dorsal frontal cortex, inferior parietal lobule, precuneus, anterior prefrontal cortex, lateral cerebellum, temporal lobe, fusiform gyrus, mid insula, etc. in order of discriminant ability. The findings are consistent with previous studies (Nickl-Jockschat et al., 2012; Hashem et al., 2020; Lau et al., 2020). We visualize the ROIs using the Brainmesh of Ch2 with Cerebellum in Figure 7, where the size of node spheres depends on the original value in the node file provided by the Dosenbach 160 template.

### Other Distribution Priors

As described before, we first give an equivalent probability explanation for PC by introducing a normal distribution for the rs-fMRI signal values. Then we reformulate PC with Bayesian rule, thus getting two perspectives of PC. This provides a platform for generalizing PC to CC by assuming that the edge weight matrix **W** follows the MVN distribution prior. As a result, we derive a probabilistic explanation of CC and develop a high-order BFN estimation framework that allows the introduction of different priors (or regularizers).

Besides the introduced normal distribution prior on **W** for BFN estimation, we can also introduce other priors on **W**. For example, considering Laplacian distribution prior for *w*_*ij*_, e.g., $P\left(w_{ij}\right)=\frac{1}{2\beta }e^{-\frac{\left|w_{ij}\right|}{\beta }}$ where β is a scale parameter. In this way, the regularized least square problem is


(24) $$\min_{w_{ij}}\sum_{ij}\left({||{\text{x}}_{i}-w_{ij}{\text{x}}_{j}||}^{2}+\lambda \left|w_{ij}\right|\right)$$

We can find that the Laplacian distribution generates sparse BFN due to the regularizer *w*_*ij*_. Besides, we can get the optimal solution by the soft thresholding, as follows:


(25) $${\hat{w}}_{ij}=\left\{ \begin{array}{cc} {\text{x}}_{i}^{T}{\text{x}}_{j}-\lambda , & {\text{x}}_{i}^{T}{\text{x}}_{j}\geq \lambda \\ 0, & {\text{x}}_{i}^{T}{\text{x}}_{j}<\lambda \end{array}\right.$$

Although different prior distributions can be tried to introduce the proposed probabilistic framework, we do not formulate their models in detail since this paper focuses on the formulation of CC.

## Conclusion

In this paper, we propose a probabilistic high-order BFN learning framework with a matrix normal penalty for ASD identification. As pointed out previously, CC is intuitively defined based on two sequential PC operations and falls short of a rigorous mathematical basis. To address this issue, we first reformulate PC with Bayesian rule and then generalize PC to CC by assuming that the edge weight matrix follows a matrix-variate normal distribution prior. This work lays the theoretical foundation for CC methods, leading to a better understanding of high-order BFN learning. In this base, we develop a Bayesian High-order Model to simultaneously estimate the high- and low-order BFN. To efficiently solve the proposed objective function, an alternating optimization algorithm is proposed. Extensive experiments on the NYU site of ABIDE dataset demonstrate the effectiveness of the proposed method, in comparison to the baseline methods. Especially for the BHM-**Ω**, it achieves the best performance. Note that we only construct high-order BFN based on PC. In principle, any correlation-based BFN estimation method (e.g., SR) can be embedded in the proposed probabilistic framework. In the future, we plan to validate the scheme on the other correlation-based BFN models.

## Data Availability Statement

The original contributions presented in the study are included in the article/supplementary material, further inquiries can be directed to the corresponding authors.

## Author Contributions

LQ proposed the idea of Bayesian high-order model. RD steered the structure of the manuscript and polished the language. XJ validated the models’ derivation, coded and performed the experiments, and planned and wrote the manuscript. YuZ set the procedures of ASD identification experiments. YiZ and LZ designed a core derivation of the models. All authors developed the estimation algorithm and contributed to the preparation of the manuscript.

## Conflict of Interest

The authors declare that the research was conducted in the absence of any commercial or financial relationships that could be construed as a potential conflict of interest.

## Publisher’s Note

All claims expressed in this article are solely those of the authors and do not necessarily represent those of their affiliated organizations, or those of the publisher, the editors and the reviewers. Any product that may be evaluated in this article, or claim that may be made by its manufacturer, is not guaranteed or endorsed by the publisher.

## Footnotes

1 Note that the rs-fMRI signals have been preprocessed as described in Section “Data Acquisitions and Processing.”

2
http://fcon_1000.projects.nitrc.org/indi/abide/

3
http://rfmri.org/dpabi

## Appendix

This Appendix consists of three appendices: Appendix A gives a detailed explanation of Eq. 9, Appendix B presents a detail for Eq. 12 and Appendix C gives a detailed derivation of Eq. 18. To keep the process of derivation smooth, we write the formulas that appeared above with the original number in the appendix, while the new formulas in the process of derivation was renumbered.

### Appendix A

Given *x*_*kj*_ and *a*_*ij*_ are constants, *x*_*ki*_ follows the normal distribution *x*_*ki*_∼𝒩(*a*_*ij*_*x*_*kj*_, σ^2^). The conditional distribution can be written as

(A1′)
$$P\left(x_{ki}|a_{ij},x_{kj},\sigma^{2}\right)=\frac{1}{\sqrt{2\pi }\sigma }e^{-\frac{{\left(x_{ki}-a_{ij}x_{kj}\right)}^{2}}{2\sigma^{2}}}$$

Assuming that the variables *x*_*ki*_ of rs-fMRI time series **x**_*i*_, *i* = 1, ⋯, *p* are independent identically distributed, the likelihood function can be written as follows:

(A2′)
$$\begin{array}{c} P\left({\text{x}}_{i}|a_{ij},{\text{x}}_{j},\sigma^{2}\right)=\prod_{k=1}^{n}P\left(x_{ki}|a_{ij},x_{kj},\sigma^{2}\right)\\ \;={\left(\sqrt{2\pi }\sigma \right)}^{-n}e^{-\frac{{||x_{i}-a_{ij}x_{j}||}^{2}}{2\sigma^{2}}} \end{array}$$

To avoid overflow caused by multiplying operations in Eq. A2′, we use the log-likelihood function and further maximize it:

(A3′)
$$\max_{a_{ij}}\text{log}P\left({\text{x}}_{i}|a_{ij},{\text{x}}_{j},\sigma^{2}\right)=\max_{a_{ij}}-n\text{log}\left(\sqrt{2\pi }\right)-\frac{{||{\text{x}}_{i}-a_{ij}{\text{x}}_{j}||}^{2}}{2\sigma^{2}}$$

### Appendix B

The edge weight *w*_*ij*_ of BFN has the following prior probabilistic density:

(A11)
P(wij)=12πe-wij22

According to Bayesian rule, the posterior distribution of *w*_*ij*_ is proportional to *P*(**x**_*i*_|*w*_*ij*_, **x**_*j*_)*P*(*w*_*ij*_):

(A4′)
$$P\left(w_{ij}|{\text{x}}_{i},{\text{x}}_{j}\right)\propto P\left({\text{x}}_{i}|w_{ij},{\text{x}}_{j}\right)P\left(w_{ij}\right)$$

where *P*(**x**_*i*_|*w*_*ij*_, **x**_*j*_) is the likelihood of *w*_*ij*_ for **x**_*i*_. Based on Eqs A2′, 11

(A5′)
P(xi|wij,xj)P(wij)=(2π)-(n+1)σ-ne-||xi-wijxj||22σ-wij22

Taking the logarithm on Eq. A4′, we obtain

(A6′)
logP(wij|xi,xj,σ2)∝-(n=1)log(2π)=(-n)logσ-||xi-wijxj||22σ2-wij22

Next, the log-posterior probability is maximized (i.e., maximal posterior estimation) as follows,

(A12)
maxwij-(n=1)log(2π)=(-n)logσ-||xi-wijxj||2=σ2wij22σ2

### Appendix C

As shown in Section “Learning High-Order Brain Functional Network With a Matrix-Normal Penalty,” the proposed model is formulated as

(A18)
$$J\left(W,\Omega \right)=\min_{W,\Omega }{||\text{W}-{\text{X}}^{T}\text{X}||}_{F}^{2}+\lambda \left[\frac{1}{2}tr\left(\Omega^{-1}\text{W}\Omega^{-1}{\text{W}}^{T}\right)+p\log\left(\left|\Omega \right|\right)\right]$$

As described before, we assume that any time series **x**_*i*_ from the data set **X** = [**x**_1_, **x**_2_, ⋯, **x**_*p*_]^*T*^ follows the conditional distribution as:

(A2′)
$$\begin{array}{c} P\left({\text{x}}_{i}|a_{ij},{\text{x}}_{j},\sigma^{2}\right)=\prod_{k=1}^{n}P\left(x_{ki}|a_{ij},x_{kj},\sigma^{2}\right)\\ \;={\left(\sqrt{2\pi }\sigma \right)}^{-n}e^{-\frac{{||x_{i}-a_{ij}x_{j}||}^{2}}{2\sigma^{2}}} \end{array}$$

Using the maximum likelihood estimation for *a*_*ij*_, we get $a_{ij}={\text{x}}_{i}^{T}{\text{x}}_{j}=w_{ij}$. Therefore, we rewrite Eq. A2′ as follows.

(A7′)
$$P\left({\text{x}}_{i}|w_{ij},{\text{x}}_{j},\sigma^{2}\right)={\left(\sqrt{2\pi }\sigma \right)}^{-n}e^{-\frac{{||x_{i}-w_{ij}x_{j}||}^{2}}{2\sigma^{2}}}$$

We can further convert Eq. A7′ to the matrix form:

(A8′)
$$P\left(\text{X}|\text{W}\right)=\prod_{i,j=1}^{p}P\left({\text{x}}_{i}|w_{ij},{\text{x}}_{j},\sigma^{2}\right)={\left(\sqrt{2\pi }\sigma \right)}^{-p^{2}n}e^{-\frac{\sum_{i,j=1}^{p}{||x_{i}-w_{ij}x_{j}||}^{2}}{2\sigma^{2}}}$$

Furthermore, as mentioned in Section “Learning High-Order Brain Functional Network With a Matrix-Normal Penalty,” we assume that the low-order correlation matrix **W** follows the MVN distribution with the probabilistic density

(A9′)
$$P\left(W\right)={\left(2\pi \right)}^{-\frac{1}{2}p^{2}}{\left|\Omega \right|}^{-p}e^{tr\left\{-\frac{1}{2}\Omega^{-1}W\Omega^{-1}W^{T}\right\}}$$

Note that the row\column variance matrix **Ω** is considered as the high-order correlation matrix between **x**_*i*_ since it models the relationships between *w*_*ij*_.

Based on the Bayesian rule, the posterior probability of **W** is given by

(A10′)
P(W|X)∝P(X|W)⋅P(W)

From Eqs A8′, A9′, we obtain

(A11′)
$$P\left(\text{X}|\text{W}\right)\cdot P\left(\text{W}\right)={\left(2\pi \right)}^{\frac{-p^{2}\left(n=1\right)}{2}}\sigma^{-p^{2}n}|\Omega {|}^{-p}\cdot e^{\text{tr}\left\{-\frac{1}{2}\Omega^{-1}W\Omega^{-1}W^{T}\right\}-\frac{\sum_{i,j=1}^{p}{||x_{i}-w_{ij}x_{j}||}^{2}}{2\sigma^{2}}}$$

Taking the logarithm of the above likelihood function and then maximizing it, we get

(A12′)
$$\max_{W,\Omega }-\frac{\sum_{i,j=1}^{p}{||{\text{x}}_{i}-w_{ij}{\text{x}}_{j}||}^{2}}{2\sigma^{2}}=tr\left\{-\frac{1}{2}\Omega^{-1}\text{W}\Omega^{-1}{\text{W}}^{T}\right\}-p\log\left(\left|\Omega \right|\right)$$

The above is equivalent to the optimization problem:

(A13′)
$$\underset{W,\Omega }{\text{min}}\frac{\sum_{i,j=1}^{p}{||{\text{x}}_{i}-w_{ij}{\text{x}}_{j}||}^{2}}{2\sigma^{2}}=tr\left\{\frac{1}{2}\Omega^{-1}\text{W}\Omega^{-1}{\text{W}}^{T}\right\}=p\log\left(\left|\Omega \right|\right)$$

Consider that the solution of the former is wij=xiTxj. Note that $\min_{w_{ij}}\sum_{i,j=1}^{p}{||{\text{x}}_{i}-w_{ij}{\text{x}}_{j}||}^{2}$ can be rewritten in matrix form as $\min_{W}{||\text{W}-{\text{X}}^{T}\text{X}||}_{F}^{2}$. Therefore, problem (A13′) can be transformed as

(A14′)
$$\min_{W,\Omega }||\text{W}-{\text{X}}^{T}\text{X}|{|}_{F}^{2}=\lambda \left[\frac{1}{2}tr\left(\Omega^{-1}\text{W}\Omega^{-1}{\text{W}}^{T}\right)=p\text{log}\left(|\Omega |\right)\right]$$
